# The Colorectal Cancer Glycocode: Tumour Sialylation Is Associated with an Immune-Excluded Phenotype and Distinct Therapeutic Signatures

**DOI:** 10.3390/biology15090705

**Published:** 2026-04-30

**Authors:** Abdulaziz Alfahed, Glowi Alasiri, Abdulrahman A. Alahmari

**Affiliations:** 1 Department of Medical Laboratory, College of Applied Medical Sciences, Prince Sattam Bin Abdulaziz University, AlKharj 11942, Saudi Arabia; aa.alahmari@psau.edu.sa; 2 Department of Biochemistry, College of Medicine, Imam Mohammad Ibn Saud Islamic University (IMSIU), Riyadh 13317, Saudi Arabia; gaalasiri@imamu.edu.sa

**Keywords:** sialylome, glycobiology, colorectal cancer, glyco-immune checkpoint, Siglec signalling, immune exclusion, epithelial–mesenchymal transition (EMT), vesicular trafficking, multidrug resistance, therapeutic vulnerability

## Abstract

Background: Tumour glycosylation regulates immune modulation and progression, but whether the CRC sialylome—the complete repertoire of sialylated glycans—defines a biologically distinct subtype remains unclear. We investigated how the “sugar code” shapes CRC biology, immunity, and therapeutic response. Methods: Transcriptomic data from three CRC cohorts (TCGA, Sidra-LUMC, and CPTAC-2; n = 988) were batch-corrected and integrated. Single-sample gene set enrichment analysis (ssGSEA) quantified sialyltransferase expression, sialic acid metabolism, EMT, MDR mechanisms, immune phenotypes, and Siglec-associated transcriptional signatures. GSEA, gene ontology enrichment analysis (GOEA), and drug ontology enrichment analysis (DOEA) characterised pathways and identified drug response-associated transcriptional signatures. Results: High sialylome activity defined a genomically stable but clinically advanced CRC subset enriched for left-sided tumours, mucinous histology, MSI, and *BRAF* mutations. At the transcriptional level, Sialyl-High tumours were associated with a mesenchymal, stromal-remodelling programme accompanied by reduced proliferative activity. They demonstrated enrichment of vesicular trafficking-related pathways alongside reduced representation of canonical efflux-associated programmes. Critically, the sialylome was associated with Siglec-related immune signatures, with sialylated glycan-related gene expression correlating with Siglec receptor expression (CD33 and SIGLEC7/9/10), consistent with an immune-inflamed yet structurally excluded microenvironment. DOEA identified selective enrichment of drug-response signatures related to sialic acid metabolism inhibitors (oseltamivir and Neu5Ac) and glycocalyx-disrupting agents (ginsenosides and soyasaponins). Conclusions: The CRC sialylome is associated with tumour phenotypic variation, including immune-excluded states linked to Siglec-associated transcriptional signatures and patterns consistent with non-canonical drug resistance programmes. These findings position the “sugar code” as a central organising principle in CRC and identify glycan-directed therapies as a promising strategy for the targeting of this aggressive subtype.

## Simple Summary

Our cells are covered in a dense layer of sugar molecules called glycans, which form a unique “sugar code” that influences how cells behave and interact. In cancer, this sugar code becomes corrupted. In this study, we investigated how changes in one type of sugar modification—called sialylation—affects colorectal cancer progression, immune evasion, and treatment response. Analysing nearly 1000 tumour samples, we discovered that some colorectal cancers have a dramatically altered “sugar coat.” These sugar-coated tumours are more aggressive, spread more easily, and adopt a “mesenchymal” identity that helps them migrate. Importantly, their sugary surface acts as a camouflage, engaging “docking stations” (called Siglecs) on immune cells and effectively telling the immune system to “stay away”. This creates a tumour microenvironment where immune cells are present but functionally excluded—they can see the cancer but cannot attack it. We also found that these sugar-coated tumours resist chemotherapy through a novel mechanism: they trap drugs inside cellular compartments rather than pumping them out. However, this unique sugar biology also creates a vulnerability. Our analysis suggests these tumours may be susceptible to drugs that interfere with sugar metabolism or strip away their sugary camouflage. This study reveals that the “sugar code” is not just a passive feature but an active driver of cancer behaviour, opening new avenues for therapies that target tumour glycosylation.

## 1. Introduction

Colorectal cancer (CRC) remains one of the most common malignancies worldwide and a leading cause of cancer-related mortality, with approximately 1.9 million new cases and 900,000 deaths annually [1]. Despite significant advances in screening, surgery, and multimodal therapies, a substantial proportion of patients develop metastatic disease or exhibit therapeutic resistance. This persistent clinical challenge underscores the urgent need to move beyond conventional genomic and transcriptomic classifications and explore other layers of molecular complexity that drive tumour heterogeneity, progression, and treatment failure. Among these, the cancer glycome—the complete repertoire of glycan structures expressed by tumour cells—has emerged as a critical but historically understudied determinant of malignant behaviour [2,3].

Glycobiology, the study of glycan structures and their functions, has undergone a renaissance in recent decades, driven by technological advances in glycomics, glycoproteomics, and analytical chemistry. It is now firmly established that glycans are not merely structural components of the cell surface or inert metabolic byproducts [2]. Rather, they are active and dynamic participants in virtually all physiological and pathological processes. Glycans mediate protein–protein and protein–carbohydrate interactions, modulate cell signalling pathways by influencing receptor conformation and clustering, regulate immune-cell recognition through “self-associated molecular patterns”, and serve as critical interface molecules in host–pathogen interactions [4,5]. In cancer, the glycome is profoundly altered, and these changes are not epiphenomena but functional drivers of tumour progression, metastasis, immune evasion, and therapeutic response [3,6].

Among the most common and functionally significant glycan modifications in cancer is sialylation—the enzymatic addition of sialic acid residues to the termini of glycan chains on glycoproteins and glycolipids [4,6]. This modification is catalysed by a family of sialyltransferases (principally, the ST3GAL, ST6GAL, and ST8SIA families), which add sialic acids in specific glycosidic linkages (α2–3, α2–6, and α2–8). The expression of these enzymes is frequently dysregulated in cancer, leading to a remodelled sialylome—the complete set of sialylated structures in a cell—which profoundly alters tumour biology [7,8]. The functional consequences of an altered sialylome are manifold. Sialylation of receptor tyrosine kinases (e.g., EGFR, MET, and HER2) can enhance ligand-independent activation and downstream signalling through MAPK and PI3K/AKT pathways, thereby promoting proliferation and survival [5,9]. Sialylated glycans on adhesion molecules (e.g., integrins and cadherins) modulate cell–cell and cell–matrix interactions, facilitating detachment from the primary tumour and invasion into surrounding tissues—key steps in the metastatic cascade [7,8]. Furthermore, the dense array of sialic acids on the tumour cell surface contributes to the formation of a glycocalyx, a carbohydrate-rich layer that can physically shield tumour cells from immune recognition and physically constrain immune synapse formation [2,4].

Perhaps the most clinically significant function of tumour sialylation lies in its ability to directly regulate anti-tumour immunity. Sialic acids are recognised by a family of inhibitory receptors called Siglecs (sialic acid-binding immunoglobulin-type lectins), which are expressed predominantly on immune cells, particularly those of the myeloid lineage (e.g., macrophages, neutrophils, and dendritic cells) [10,11]. Engagement of these receptors by tumour-associated sialylated glycans delivers inhibitory signals that dampen immune-cell activation, promote a tolerogenic phenotype, and effectively establish a glyco-immune checkpoint [12,13]. This axis has been implicated in creating “immune-excluded” or “immune-desert” tumour microenvironments, where immune cells are either physically barred from engaging tumour cells or functionally silenced upon arrival [2,14]. In CRC, emerging evidence suggests that sialylation of immune checkpoint molecules, themselves, such as PD-L1, can stabilise the protein and enhance its immunosuppressive function, further compounding immune evasion [8].

Beyond immune modulation, the tumour glycome is intimately linked with other core cancer programmes. Epithelial–mesenchymal transition (EMT), a process by which epithelial cells acquire mesenchymal traits of motility and invasion, is accompanied by profound remodelling of the cell-surface glycome [15,16]. EMT transcription factors can directly regulate the expression of glycosyltransferases, and the resulting changes in glycan structures on cadherins and integrins are functionally required for the completion of the EMT programme [16]. Similarly, altered glycosylation has been linked to multidrug resistance (MDR), though the mechanisms remain poorly defined. While classical MDR is often attributed to drug efflux pumps (e.g., P-glycoprotein), emerging evidence suggests that glycosylation may influence drug handling through effects on drug uptake, intracellular trafficking, and sequestration within the endomembrane system—the very cellular compartment where glycosylation, itself, occurs [17,18,19].

Although prior studies have highlighted the biological relevance of sialylation in cancer, most have been limited to small cohorts, focused on single genes or glycan structures, or restricted to in vitro models. A comprehensive, systems-level analysis that integrates the sialylome with EMT, MDR, immune architecture, and therapeutic response across large, well-annotated CRC cohorts is lacking [7,8]. Critically, it remains unknown whether tumour sialylation defines a biologically and therapeutically distinct CRC subset, that is, whether the “sugar code” can be used to stratify patients and guide treatment decisions. Addressing this gap requires a move beyond reductionist approaches to embrace the complexity of glycan-mediated biology.

To address these knowledge gaps, we analysed transcriptomic data from three independent CRC cohorts—TCGA, Sidra-LUMC, and CPTAC-2—using a glycobiology-centred analytical framework integrating single-sample gene set enrichment analysis (ssGSEA), gene set enrichment analysis (GSEA), gene ontology enrichment analysis (GOEA), and drug ontology enrichment analysis (DOEA) [20,21,22,23,24,25]. In this study, we aimed to determine whether the tumour sialylome defines a distinct CRC subset with unique biological and therapeutic properties. Specifically, we sought to: (i) characterise the sialylome of CRC by quantifying the coordinated expression of sialyltransferases, sialic acid metabolism enzymes, and related glycan-modifying genes and to link this sialylome to specific clinicopathological and molecular features; (ii) determine how the sialylome integrates with core cancer programmes, including EMT, ECM remodelling, proliferation, and MDR, to test the hypothesis that glycosylation is a central organising principle in the CRC phenotype [26,27,28,29]; (iii) decode the glyco-immune interface by investigating the relationship between tumour sialylation and immune architecture (inflamed, excluded, and desert phenotypes),and by quantifying Siglec-associated transcriptional signatures using composite Siglec scores and pathway analyses [10,11,14,29,30]; and (iv) identify drug response-associated transcriptional signatures linked to the Sialyl-High subtype by performing drug ontology enrichment analysis on the genes driving glycan-associated programmes, with the aim of highlighting candidate pathways for therapeutic investigation [25].

Collectively, these analyses provide an integrated, multi-cohort framework to evaluate how the tumour sialylome shapes CRC biology, the immune microenvironment, and drug response. By placing glycosylation at the centre of our investigation, we aim to demonstrate that the “sugar code” is not merely a decorative feature of cancer cells but a systems-level determinant of tumour behaviour and a promising target for stratified therapy. This study is designed as a computational, hypothesis-generating framework and does not include experimental validation.

## 2. Materials and Methods

### 2.1. Study Cohorts and Data Integration: A Multi-Platform Glycobiological Resource

To investigate the functional landscape of the CRC glycome, transcriptomic and clinical data were obtained from three independent colorectal cancer (CRC) cohorts. The first cohort comprised samples from The Cancer Genome Atlas Colorectal Adenocarcinoma (TCGA-COADREAD) project, which provides harmonised RNA-sequencing data with detailed clinicopathological annotation [20]. The second cohort was the Sidra-LUMC CRC cohort, a clinically annotated series with integrated transcriptomic and genomic profiling [21]. The third cohort consisted of CRC cases from the Clinical Proteomic Tumor Analysis Consortium phase 2 (CPTAC-2), which offers complementary proteogenomic and transcriptomic data [22]. To create a unified, high-powered discovery dataset for glycomic analysis, the gene expression matrices from all three cohorts were integrated. Gene Ensembl IDs from the TCGA matrix were first converted to gene symbols by mapping to the Molecular Signature Database (MSigDB) Human ENSEMBL-Gene ID version 7.0 chip [23]. The matrices were then filtered to retain only genes common to all three cohorts (n = 11,731). To mitigate technical batch effects arising from different sequencing platforms and processing pipelines while preserving true biological variation in glycan-related gene expression, the ComBat algorithm was applied [31]. ComBat was implemented as a Bash-executable R script on Ubuntu 20.04, producing a single, harmonised expression matrix. Principal component analysis (PCA) was used to confirm the effective removal of cohort-specific variance, ensuring that subsequent analyses would reflect genuine tumour biology.

### 2.2. Deconvoluting the CRC Sialylome: Derivation of Functional Glycosylation Scores

To move beyond a single marker and capture the systems-level activity of the glycosylation machinery, we used single-sample gene set enrichment analysis (ssGSEA) with custom R scripts to derive multiple functional indices from the integrated expression matrix [26,27]. This approach allows us to quantify the coordinated activity of entire biological pathways. Specifically, we quantified the following aspects of the tumour glycome and its functional context:

(i) The Tumour Sialylome: A composite “sialylation activity” score was derived using a curated gene set encompassing the key components of the biosynthetic and conjugative machinery of sialic acid. This gene set comprehensively included sialyltransferases from the ST3GAL, ST6GAL, ST6GALNAC, and ST8SIA families responsible for adding sialic acids in specific glycosidic linkages. Specifically, the ST3GAL family was represented by ST3GAL1, ST3GAL2, ST3GAL3, ST3GAL4, ST3GAL5, and ST3GAL6. The ST6GAL family was represented by ST6GAL1 and ST6GAL2, and the ST6GALNAC family, responsible for O-glycan sialylation, was comprehensively covered by ST6GALNAC1, ST6GALNAC2, ST6GALNAC3, ST6GALNAC4, ST6GALNAC5, and ST6GALNAC6. The ST8SIA family, which synthesizes polysialic acids, was represented by ST8SIA1, ST8SIA2, ST8SIA3, ST8SIA4, ST8SIA5, and ST8SIA6 [2,4].
(ii) Glycan-Associated Functional Programmes: To understand the downstream consequences of an altered glycome, we quantified programmes known to be intimately linked with glycosylation, including epithelial–mesenchymal transition (EMT), stromal remodelling, and proliferation, using validated gene signatures previously applied in CRC and pan-cancer analyses [28,29].
(iii) Multidrug Resistance (MDR) Mechanisms: To test the hypothesis that the glycome influences drug handling, we quantified seven distinct MDR programmes—drug efflux, metabolic inactivation, apoptosis suppression, target bypass signalling, stress adaptation, xenobiotic sensing, and xenobiotic trafficking and sequestration—using established gene sets [17,18]. This allows us to determine which resistance mechanisms are preferentially enriched in association with a given glycosylation state.
(iv) The Glyco-Immune Interface: We assessed the tumour immune landscape using established signatures for immune-inflamed, immune-excluded, and immune-desert phenotypes [14,30]. Furthermore, to directly probe the interaction between tumour glycans and immune cells, we derived a Siglec score, which was calculated as the combined expression of CD33, SIGLEC7, SIGLEC9, and SIGLEC10. This score serves as a transcriptomic proxy for the involvement of the sialic acid–Siglec immunoregulatory axis, reflecting the presence of myeloid and other immune cells bearing receptors for sialylated glycans [10,11].

The exact compositions of all gene lists used to generate these functional scores are available in Supplementary Materials Table S1.

### 2.3. Pathway and Ontology Enrichment Analyses: Decoding the Functional Context of the Glycome

To elucidate the biological programmes and drug response-associated transcriptional signatures linked to the CRC glycome, we performed several complementary enrichment analyses: (i) Gene Set Enrichment Analysis (GSEA): To identify differentially enriched pathways between high- and low-sialylation tumours, we used MSigDB GSEA software version 4.3.3 with the Hallmark, Reactome, and Gene Ontology gene-set collections [24]. This provides a broad, unbiased view of the biological processes linked to the glycosylation state. (ii) Gene Ontology Enrichment Analysis (GOEA) and Drug Ontology Enrichment Analysis (DOEA): To perform more detailed and exploratory enrichment analyses, we used Enrichr (https://maayanlab.cloud/Enrichr/, accessed on 26 February 2026) [25]. For DOEA, we focused on genes driving the core sialylation, ECM, and Siglec programmes to identify compounds with known response signatures that may suggest candidate pathways for therapeutic exploration in the Sialyl-High subtype. Drug ontology enrichment analysis identifies associations between gene expression patterns and known drug-response signatures; it does not provide evidence of drug efficacy, dose–response relationships, or clinical applicability.

### 2.4. Statistical Analyses and Figure Generation

All statistical analyses were performed to test the central hypothesis that the tumour sialylome defines a distinct biological state. Associations between the dichotomised sialylation index (high vs. low) and clinicopathological or molecular features were assessed using chi-square tests for categorical variables. Continuous ssGSEA-derived scores (e.g., EMT, Siglec, and MDR scores) were compared between groups using Mann–Whitney U and Kruskal–Wallis tests, with Spearman’s rank correlation used to assess continuous associations between glycan-related scores and other functional indices. To determine whether the association between sialylation and immune exclusion was independent of other immune phenotypes, we used multivariable linear regression models. To control for multiple-hypothesis testing, the Benjamini–Hochberg procedure was applied, with an adjusted *p* < 0.05 considered statistically significant. All figures were generated using SPSS v29 and the SRplot platform (https://www.bioinformatics.com.cn/en, accessed on 27 February 2026) [32].

### 2.5. Differential Expression Analysis

Differential expression analysis was performed to identify gene-level transcriptional differences between Sialyl-High and Sialyl-Low tumours and to complement the pathway-level analyses. The analysis was conducted using a linear modelling framework with empirical Bayes moderation, as implemented in the limma package. This approach is appropriate for pre-normalised expression data and was applied to the batch-corrected expression matrix.

## 3. Results

A sialylation signature, encompassing genes for sialyltransferases (e.g., ST3GAL and ST6GAL families) was quantified in 988 CRC cases using ssGSEA. The median score was 1.081 (range 0.401–1.798). To enrich for a robust glycosylation phenotype, cases were dichotomised at the 67th percentile into “low sialylation” (Sialyl-Low) and “high sialylation” (Sialyl-High) subsets. Validation using the Reactome Pathway library confirmed that the Sialyl-High subset was genuinely enriched for core glycobiology pathways: glycosylation, sialic acid metabolism, N-/O-glycan biosynthesis, and ECM proteoglycan interactions (Supplementary Materials Table S2). This confirms that our index captures a distinct state of the tumour glycome.

### 3.1. The CRC Sialylome Is Associated with Distinct Clinicopathologic and Genomic Contexts

We first investigated how the tumour sialylome aligns with clinical and molecular features (Supplementary Materials Table S3, Table 1). High sialylation was significantly associated with left-sided tumours, mucinous adenocarcinoma histology, and advanced overall stage (III–IV). From a glycobiology perspective, the link with mucinous histology is particularly salient, as these tumours are defined by their abundant, heavily O-glycosylated mucin production, representing an extreme example of glycan-directed tumour biology. Genomically, the Sialyl-High subset was enriched for microsatellite instability (MSI), hypermutated/MSI and genome-stable molecular subtypes, *BRAF* mutations, and *TP53* wild-type status. Conversely, these tumours exhibited significantly lower genomic instability metrics (Aneuploidy and Fraction Genome Altered scores). This suggests that the Sialyl-High phenotype represents a genomically stable but epigenetically/transcriptionally reprogrammed state focused on glycan biosynthesis rather than a proliferation-driven, genomically unstable tumour. No associations were found with age, sex, or survival, indicating that the sialylome defines a biological state independent of these conventional prognostic factors.

### 3.2. The Sialylome Defines a Dichotomous Tumour State: Invasive/Remodelling vs. Proliferative

To decode the functional programmes associated with the CRC sialylome, we performed GSEA comparing Sialyl-High vs. Sialyl-Low tumours.

#### 3.2.1. The Sialyl-High Glycophenotype Is Inflammatory and Stromal-Interactive

Sialyl-High tumours were consistently enriched for hallmark gene sets associated with inflammation (TNFα/NF-κB, IL6–JAK–STAT3, and IFNγ response), hypoxia, angiogenesis, and KRAS signalling (FDR < 0.25, nominal *p* < 0.05; Supplementary Materials Table S4). Critically, from a glycobiology standpoint, these tumours showed significant enrichment for Epithelial–Mesenchymal Transition (EMT) and pathways related to extracellular matrix (ECM) organisation. GOEA further refined this, revealing enrichment for “extracellular matrix organization”, “collagen formation”, “integrin signalling”, and “glycosaminoglycan metabolism” (adjusted *p* < 0.05; Supplementary Materials Table S5). This paints a picture of a tumour that is not just passively glycosylated but that actively uses its glycan coat to interact with and remodel its surrounding microenvironment. The enrichment of “platelet activation” and “GPCR signalling” pathways also suggests that the sialylated glycocalyx may engage with receptors on stromal and immune cells.

#### 3.2.2. The Sialyl-Low Glycophenotype Is Proliferative and Replicative

**Figure 1:**
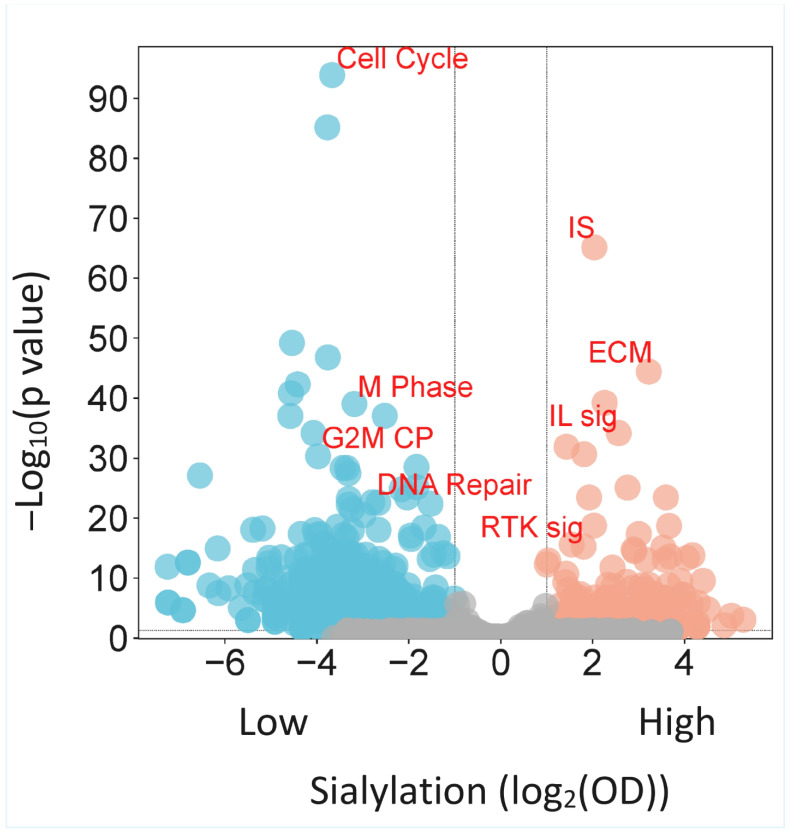
Volcano plot revealing the functional landscape of the CRC sialylome. Ontology terms enriched in the Sialyl-High subset (red) reflect glycan-associated programmes including immune modulation and ECM remodelling, while terms enriched in Sialyl-Low tumours (blue) reflect proliferative, replication-driven biology. This dichotomy illustrates how the tumour glycome dictates divergent functional states. Grey dots represent non-significant genes. IS = immune system; IL sig= interleukin signalling; ECM = extracellular matrix; RTK sig = receptor tyrosine kinase signalling; CP = checkpoint. The dotted line denotes the threshold of significance for enrichment of the ontology terms.

In stark contrast, Sialyl-Low tumours were exclusively enriched for proliferative programmes: MYC/E2F targets, G2M checkpoint, DNA replication, and DNA repair pathways (FDR < 0.25, nominal *p* < 0.05; Supplementary Materials Table S6). GOEA confirmed this with terms like “cell cycle”, “DNA replication”, and “mitotic spindle checkpoint” (adjusted *p* < 0.05; Supplementary Materials Table S7). This dichotomy reveals a fundamental trade-off in tumour biology: the allocation of cellular resources towards the building of a complex, signalling-competent glycocalyx and interaction with the microenvironment (Sialyl-High) versus rapid cell division and replication (Sialyl-Low). This trade-off is visually summarised in the volcano plot in Figure 1, showing immune/ECM terms enriched in the high-sialylation group and cell-cycle terms in the low-sialylation group.

### 3.3. Validating the Glycan-Associated Functional Shift: EMT, Stroma, and Proliferation

**Figure 2:**
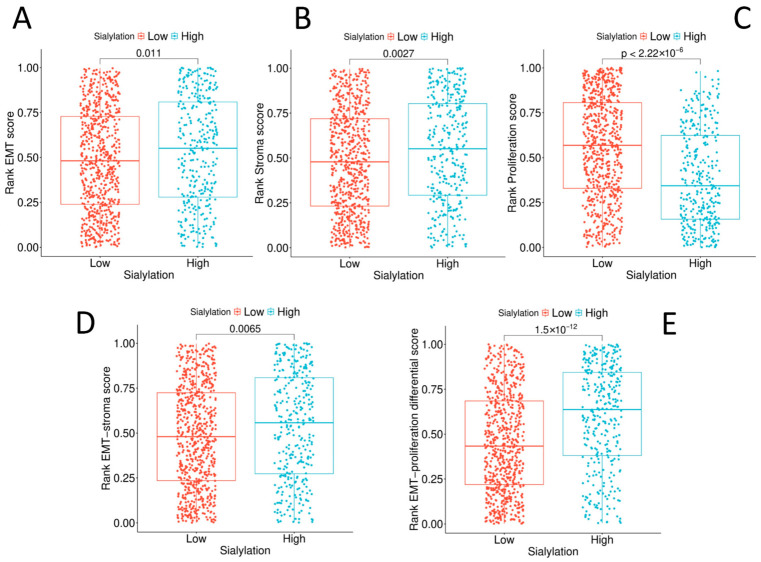
High tumour sialylation is associated with a mesenchymal–stromal phenotype and reduced proliferative activity in CRC. Box-and-jitter plots showing ssGSEA-derived rank scores for (**A**) epithelial–mesenchymal transition (EMT), (**B**) stromal signature, (**C**) proliferation, (**D**) composite EMT–stromal score, and (**E**) EMT–proliferation differential score in colorectal cancer cases stratified by tumour sialylation status (low vs. high; dichotomised at the 67th percentile). Each dot represents an individual tumour sample. Boxes indicate the interquartile range (IQR), centre lines represent the median, and whiskers extend to 1.5 × IQR. Compared with Sialylation-Low tumours, Sialylation-High tumours demonstrate significantly higher EMT (*p* = 0.011), stromal (*p* = 0.0027), EMT–stromal (*p* = 0.0065), and EMT–proliferation differential scores (*p* = 1.5 × 10^−12^) and significantly lower proliferation scores (*p* < 2.22 × 10^−16^) (Mann–Whitney U test).

To validate the GSEA findings, we derived quantitative scores for EMT, stroma, and proliferation. The sialylation index correlated positively with EMT (R = 0.130) and stromal scores (R = 0.140) and negatively with proliferation (R = −0.353). The composite “EMT–proliferation differential” score was significantly higher in the Sialyl-High subset (*p* < 0.001; Figure 2).

These results provide quantitative evidence for the model proposed above: high tumour sialylation is functionally linked to a mesenchymal, stromal-engaging phenotype at the expense of rapid proliferation. This suggests that the glycan repertoire of a tumour is intrinsically linked to its core identity along the proliferation–invasion axis.

### 3.4. The Sialylome Dictates the Mechanism of Multidrug Resistance: Vesicular Trafficking over Efflux

**Figure 3:**
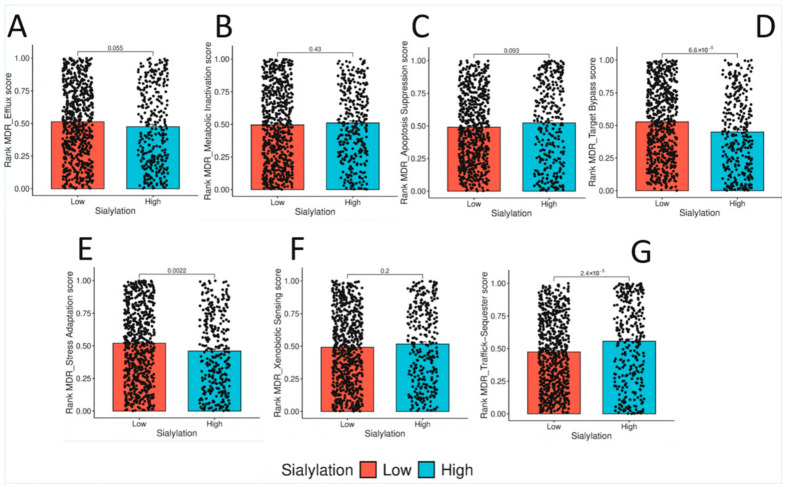
High tumour sialylation selectively associates with vesicular trafficking-mediated multidrug resistance programmes in colorectal cancer. Bar-and-jitter plots showing ssGSEA-derived rank scores for multidrug resistance (MDR) programmes in colorectal cancer cases stratified by tumour sialylation status (low vs. high; dichotomised at the 67th percentile). Panels represent: (**A**) drug efflux, (**B**) metabolic inactivation, (**C**) apoptosis suppression, (**D**) target bypass signalling, (**E**) stress adaptation, (**F**) xenobiotic sensing, and (**G**) vesicular trafficking/sequestration. Each dot represents an individual tumour sample. Boxes indicate the interquartile range (IQR), centre lines represent the median, and whiskers extend to 1.5 × IQR. Compared with Sialylation-Low tumours, Sialylation-High tumours demonstrated significantly higher vesicular trafficking/sequestration scores (*p* = 2.4 × 10^−5^) and lower stress adaptation scores (*p* = 0.0022), whereas efflux (*p* = 0.055), metabolic inactivation (*p* = 0.43), apoptosis suppression (*p* = 0.093), xenobiotic sensing (*p* = 0.2), and target bypass signalling (*p* = 6.6 × 10^−5^) showed variable or non-dominant associations (Mann–Whitney U test).

Given the link between EMT and drug resistance, we investigated which MDR programmes were associated with the Sialyl-High tumours. We analysed seven MDR mechanisms and found a highly selective pattern (Figure 3). Sialylation was positively correlated with vesicular trafficking and sequestration (rho = 0.213, *p* < 0.001) and negatively correlated with canonical resistance pathways like drug efflux (rho = −0.115), target bypass signalling (rho = −0.155), and stress adaptation (rho = −0.108). These findings imply that the tumour cell’s altered sialylation state may be functionally involved in intracellular membrane dynamics, potentially linking Golgi-based glycosylation processes with endomembrane vesicular trafficking. This connection further suggests that the “sugar code” could directly drive a specific, non-canonical mechanism of drug resistance. These findings suggest a potential non-canonical resistance-associated programme, which requires experimental validation.

### 3.5. Decoding the Glyco-Immune Interface: Inflammation, Exclusion, and the Siglec Axis

A central aim of glycobiology is to understand how glycans regulate immunity. Our analysis revealed a complex and nuanced glyco-immune landscape in Sialyl-High CRC.

#### 3.5.1. An Inflamed Yet Excluded Immune Microenvironment

**Figure 4:**
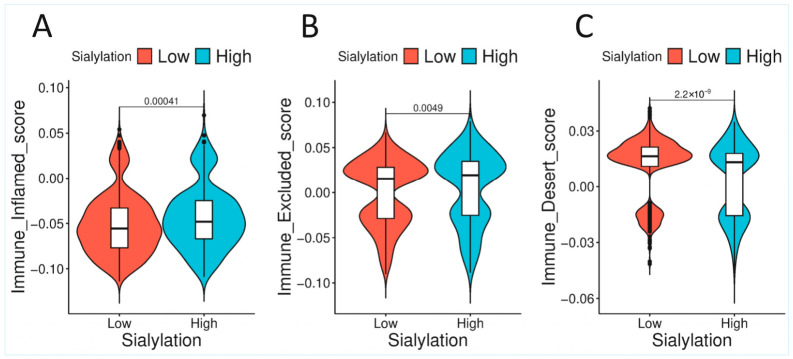
Tumour sialylation is associated with distinct immune phenotypes in CRC. Violin plots with embedded box plots showing ssGSEA-derived immune phenotype scores in CRC cases stratified by tumour sialylation status (low vs. high). (**A**). Immune-inflamed score (**B**). Immune-excluded score (**C**). Immune-desert score. White boxes represent the interquartile range (IQR), centre lines indicate the median, and whiskers extend to 1.5 × IQR. High-sialylation tumours demonstrated significantly higher immune-inflamed (*p* = 0.00041) and immune-excluded (*p* = 0.0049) scores, whereas immune-desert scores were significantly lower in Sialylation-High tumours (*p* = 2.2 × 10^−9^) (Mann–Whitney U test).

Sialyl-High tumours exhibited transcriptional evidence of immune activation, with higher scores for both “immune-inflamed” and “immune-excluded” phenotypes and a significantly lower score for the “immune-desert” phenotype (*p* < 0.001 for all comparisons; Figure 4). In multivariable analysis, the immune-excluded phenotype maintained the strongest independent association with sialylation (t = 7.016, *p* < 0.001). This indicates that while immune cells are recruited to the tumour microenvironment (inflamed), their ability to penetrate the tumour parenchyma and contact cancer cells is structurally or functionally impaired (excluded). The glycocalyx, the dense layer of sialylated glycans on the tumour cell surface, is a prime candidate for creating this physical and signalling barrier.

#### 3.5.2. The Sialic Acid–Siglec Axis and the Immune-Excluded Phenotype

**Figure 5:**
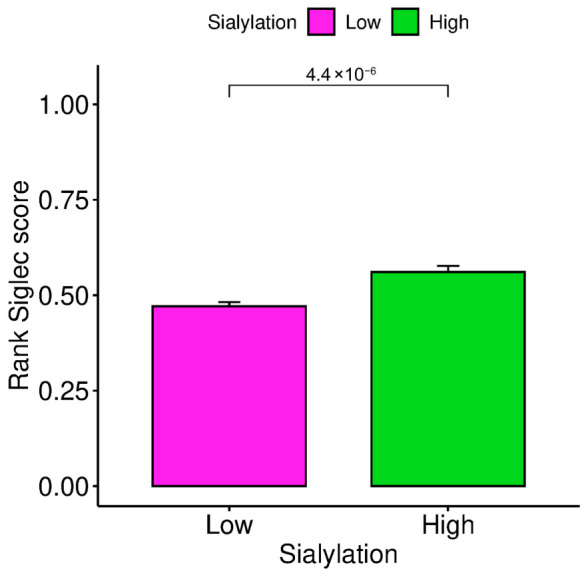
High tumour sialylation is associated with increased Siglec pathway activity in CRC. Comparison of ssGSEA-derived Siglec score between SIALYLATION-Low and Sialylation-High CRC tumours. The Siglec score was calculated as the combined expression of CD33, SIGLEC7, SIGLEC9, and SIGLEC10. Bars represent median rank scores, with error bars indicating the interquartile range (or standard error; specified as appropriate). Sialylation-High tumours demonstrated significantly higher Siglec scores compared with Sialylation-Low tumours (*p* = 4.4 × 10^−6^; Mann–Whitney U test), consistent with involvement of the sialic acid–Siglec immunoregulatory axis.

We hypothesised that the immune-excluded phenotype may be associated with Siglec-related immune regulatory pathways potentially linked to tumour sialylation. GSEA confirmed the enrichment of CD22- (Siglec-2) and CD33 (Siglec-3)-associated genes in the Sialyl-High subset (FDR < 0.25, nominal *p* < 0.05; Supplementary Materials Table S8). GOEA of these Siglec-associated genes revealed enrichment of innate immune pathways, particularly “neutrophil degranulation” and “Toll-like receptor cascades”, pointing to myeloid cells (macrophages and neutrophils) as the key immune population engaging this axis (adjusted *p* < 0.05; Supplementary Materials Table S8). There was lack of enrichment of ontology terms in the Sialyl-Low subset (Supplementary Materials Table S9). To quantify this, we derived a composite Siglec score from genes expressed in the tumour microenvironment (CD33, SIGLEC7, SIGLEC9, and SIGLEC10). This score was significantly higher in the Sialyl-High subset (*p* < 0.001; Figure 5) and correlated positively with the sialylation index itself (rho = 0.239, *p* < 0.001). These findings provide a mechanistic basis for the immune-excluded phenotype. These findings suggest that tumour sialylation is associated with increased expression of inhibitory Siglec receptors on infiltrating myeloid cells, consistent with a potential role for the sialic acid–Siglec axis in shaping an immune-excluded microenvironment. This supports the hypothesis that the ‘sugar code’ may contribute to immune regulatory processes in CRC.

### 3.6. Potential Therapeutic Implications of the CRC Sialylome: Targeting the Glycocode

Finally, we explored whether the unique glycobiology of Sialyl-High tumours is associated with distinct drug response-related transcriptional patterns. Using DOEA on the genes driving the sialylation, ECM, and Siglec programmes, we identified compounds with selectively enriched response signatures in the Sialyl-High subset. Significant enrichment was observed for the following: (i) modulators of sialic acid biology: Neu5Ac (the canonical sialic acid precursor), oseltamivir (a neuraminidase inhibitor with potential off-target effects on human sialidases); (ii) natural products that insert into membranes and modulate glycosylation: ginsenosides, soyasaponin I, and timosaponin, which are known to integrate into lipid rafts and can alter the activity of glycosyltransferases and the organisation of the glycocalyx; (iii) signalling modulators with glycan-related effects: lithium chloride (a GSK3β inhibitor that can influence glycosyltransferase gene expression) and lithocholic acid. Notably, no enrichment was seen for agents like gemtuzumab ozogamicin, indicating that the therapeutic associations are selective and tied to the specific glycobiology of this subtype (Supplementary Materials Table S10). These findings are hypothesis-generating, suggesting that Sialyl-High CRC shows enrichment of gene expression signatures associated with drugs that directly target sialic acid metabolism, disrupt the organisation of the glycocalyx, or interfere with the biosynthesis of the glycan structures that drive immune exclusion.

### 3.7. Differential Expression Confirmed Sialylation-Associated Gene Programmes

Differential expression analysis between Sialyl-High and Sialyl-Low tumours identified widespread transcriptional reprogramming, with marked upregulation of genes involved in glycan biosynthesis and epithelial secretory differentiation in the Sialyl-High subset (Supplementary Materials Table S11). Notably, key sialyltransferases (ST6GALNAC1, ST6GALNAC6, and ST3GAL4) and glycosylation-related enzymes (B3GNT6 and B4GALT4) were among the most significantly upregulated genes, confirming activation of the sialylation machinery. This was accompanied by strong enrichment of mucin-associated and secretory lineage markers, including MUC2, MUC4, FCGBP, SPDEF, and ATOH1, consistent with a goblet cell-like differentiation programme. In addition, genes associated with vesicular trafficking and secretion (e.g., RAB27A and RAB26) were significantly upregulated, supporting the enrichment of trafficking-related pathways observed in ssGSEA analyses. Conversely, Sialyl-Low tumours demonstrated relative enrichment of genes linked to proliferative and transcriptional regulatory programmes, including YAP1 and CDK8, consistent with a more proliferative phenotype. Collectively, these findings provide gene-level validation of the pathway-level analyses, demonstrating that the Sialyl-High phenotype is characterised by coordinated activation of glycosylation, secretory differentiation, and trafficking-associated programmes.

## 4. Discussion

In this comprehensive multi-cohort analysis, we show that the tumour sialylome—the complete set of sialylated glycans and the machinery that builds them—defines a biologically, immunologically, and clinically distinct subset of colorectal cancer (CRC). By integrating transcriptomic data from three independent datasets (TCGA, Sidra-LUMC, and CPTAC-2) and applying a glycobiology-centred analytical framework, we show that the CRC glycome is not merely a passive reflection of the tumour state but an active, systems-level determinant of phenotype. High tumour sialylation is associated with a coordinated programme involving specific clinicopathological features, a switch from proliferative to invasive biology, and selective enrichment of vesicular drug resistance mechanisms, consistent with an immune-excluded microenvironment via the sialic acid-Siglec axis. These findings suggest that the ‘sugar code’ may represent a central organising principle in CRC heterogeneity, with direct implications for biomarker development and glycan-targeted therapeutics [20,21,22].

Aberrant glycosylation has long been recognised as a hallmark of cancer, but its role has often been viewed as secondary to genetic drivers [2,3]. Our findings suggest a more integrated relationship by demonstrating that the sialylome is intimately integrated with—and may be closely linked to—core tumour biological programmes. The enrichment of glycosylation, sialic acid metabolism, and ECM proteoglycan pathways in the Sialyl-High subset (Supplementary Materials Table S1) confirms that these tumours have undergone fundamental glycomic reprogramming. This is consistent with coordinated glycomic reprogramming and suggests an active, coordinated shift in cellular machinery.

This reprogramming aligns with and may contribute to the observed invasive phenotype. The concurrent enrichment of EMT, ECM organisation, and integrin signalling pathways (Figure 1, Supplementary Materials Table S3) is notable from a glycobiology perspective. Sialylated glycans on integrins and other cell adhesion molecules directly modulate their conformation, clustering, and binding affinity, thereby influencing cell migration and invasion [7,8]. Furthermore, the sialylation of ECM components, themselves (collagens, laminins, and proteoglycans), can alter matrix stiffness and architecture, creating a feedback loop that further promotes a mesenchymal state [15,16]. Our data suggest that in CRC, the “sugar code” is associated with the mechanistic engine driving EMT and stromal interaction rather than constituting a downstream consequence.

The stark dichotomy between the Sialyl-High (invasive/stromal) and Sialyl-Low (proliferative) transcriptional landscapes (Section 3.2 and Section 3.3) reveals a fundamental trade-off in tumour biology: the allocation of cellular resources towards the building of a complex, signalling-competent glycocalyx versus rapid cell division. This trade-off, as visually captured by the inverse correlation between sialylation and proliferation scores (Figure 2), suggests that the glycome is a primary axis of functional divergence in CRC. This finding expands current models of tumour heterogeneity, which often focus on genomic or transcriptomic subtypes, by adding a critical layer of post-translational modification-driven diversity.

A central contribution of this study is the detailed characterisation of the glyco-immune interface in CRC. While Sialyl-High tumours exhibit transcriptional evidence of immune activation (inflamed phenotype), they are predominantly characterised by immune exclusion (Section 3.5.1, Figure 4). This paradox—immune cells present but unable to effectively engage tumour cells—is a hallmark of resistance to immunotherapy [33]. Our data point to the tumour glycocalyx as a primary architect of this barrier.

The positive correlation between sialylation and the Siglec score (Figure 5), coupled with the enrichment of CD22/CD33-associated gene sets in the Sialyl-High subset, provides a compelling mechanistic link. We propose a model where the dense array of sialylated glycans on the tumour cell surface engages inhibitory Siglec receptors on infiltrating immune cells, primarily of the myeloid lineage (as suggested by the enrichment of neutrophil degranulation and TLR pathways in GOEA). This engagement acts as a potent “don’t eat me” or “don’t migrate” signal, preventing effective anti-tumour immunity [10,11,12,13]. From a glycobiology standpoint, this is consistent with a role for the ‘sugar code’ in regulating a critical immune checkpoint. The specificity of this interaction is a key area for future investigation. Different Siglecs have preferences for different sialic acid linkages (e.g., Siglec-1 binds α2–3 and α2–6, Siglec-7 prefers α2–8 disialic acids, and Siglec-9 binds both α2–3 and α2–6) [11]. Our current transcriptomic approach cannot resolve which specific sialyltransferase (e.g., high ST3GAL vs. high ST6GAL) is responsible for generating the immunomodulatory glycan ligands in this CRC subset. Future glycomic and glycoproteomic analyses are essential to map the exact sialylated structures—such as sialyl-Lewis X and α2–6-sialylated N-glycans on specific carrier proteins like MUC1 or PD-L1—that engage specific Siglec receptors [4,8]. This structural resolution is the next frontier in decoding the glyco-immune checkpoint and designing targeted therapies. The Siglec score is derived from bulk transcriptomic data and therefore reflects the abundance of Siglec-expressing immune cells rather than direct ligand–receptor engagement, which would require spatial or protein-level validation. Bulk transcriptomic data do not provide spatial or protein-level resolution; therefore, direct interaction between tumour-associated sialylated glycans and Siglec receptors cannot be inferred. Validation using spatial transcriptomics or co-localisation-based approaches (e.g., immunofluorescence or immunohistochemistry) would be required.

The observation that Sialyl-High tumours are preferentially enriched in vesicular trafficking and sequestration mechanisms for multidrug resistance rather than classical efflux pumps (Section 3.4, Figure 3) offers a novel perspective on the link between glycosylation and chemoresistance [17,18]. This finding is biologically plausible when viewed through a glycobiology lens. The endomembrane system—comprising the endoplasmic reticulum, Golgi apparatus, endosomes, and lysosomes—is the cellular factory for both glycosylation and vesicular trafficking. The enzymes that build the glycome (glycosyltransferases) reside within this system, and the traffic of proteins and lipids through this system is essential for their modification. We hypothesise that in Sialyl-High tumours, this system is “upregulated” or “repurposed”. The increased flux of glycoproteins through the Golgi for hyper-sialylation may be accompanied by an increased capacity for endocytosis, intracellular sequestration, and lysosomal exocytosis of chemotherapeutic drugs. Essentially, the very machinery that builds the complex glycocalyx may also be co-opted to trap and expel drugs, representing a novel, glycosylation-linked resistance mechanism. This finding opens new avenues for therapeutic intervention. It suggests that drugs that disrupt vesicular trafficking (e.g., chloroquine derivatives that alkalinise lysosomes) or inhibit specific steps in glycan maturation (e.g., swainsonine and kifunensine) could synergise with conventional chemotherapy by simultaneously targeting the glycocalyx and the resistance mechanism it enables.

Drug ontology enrichment analysis (DOEA) provides an exploratory but tantalising glimpse into potential therapeutic strategies for targeting the Sialyl-High subtype. The enrichment of responses to compounds that directly modulate sialic acid biology—Neu5Ac (the sialic acid precursor) and oseltamivir (a neuraminidase inhibitor)—is particularly compelling. While oseltamivir is best known as an anti-viral, it and other neuraminidase inhibitors can affect human sialidases (NEU), potentially altering the dynamic equilibrium of sialylation on the tumour cell surface and “unmasking” tumour antigens or disrupting Siglec ligand engagement [13]. The enrichment of natural products like ginsenosides, soyasaponins, and timosaponin is also intriguing from a glycobiology perspective. These compounds are known to integrate into lipid rafts—membrane microdomains rich in glycolipids and signalling proteins—and can modulate the activity of glycosyltransferases and the organisation of the glycocalyx [34,35]. This suggests that they may exert their anti-cancer effects, in part, by disrupting the architecture and function of the tumour glycome. The lack of enrichment for agents like gemtuzumab ozogamicin (an anti-CD33 antibody–drug conjugate) highlights the selectivity of these associations. It suggests that the Sialyl-High phenotype is not simply a generic “glycan-high” state but a specific configuration with unique vulnerabilities. These findings are hypothesis-generating and underscore the need for preclinical functional studies to test whether inhibiting sialylation (e.g., with sialyltransferase inhibitors like 3Fax-peracetyl-Neu5Ac), disrupting the glycocalyx with saponins, or blocking Siglec engagement with monoclonal antibodies can effectively target this aggressive CRC subtype [12,13]. The identification of compounds such as oseltamivir is based on transcriptomic associations and should be interpreted as hypothesis-generating. Functional validation in colorectal cancer models is required to determine whether these agents have any direct or indirect therapeutic activity.

This study is designed as a computational, hypothesis-generating framework and does not include experimental validation. The study provides a powerful transcriptomic roadmap of the CRC glycome, but it also highlights the critical next steps for the field. First, the observational design precludes causal inference. The mechanistic relationships implied by our transcriptomic associations—for example, that a specific sialyltransferase is associated with immune exclusion—must be functionally validated in cellular and in vivo models using gene editing (CRISPR knockouts/knockins of specific ST3GAL, ST6GAL, or ST8SIA genes) and pharmacological inhibitors. Second—and most importantly for glycobiology—transcriptomics cannot resolve the final glycan structures. mRNA levels of sialyltransferases do not always correlate perfectly with enzyme activity, and the same enzyme can produce different products depending on the availability of acceptor substrates and the cellular context. Furthermore, transcriptomics cannot tell us which specific glycoproteins are carrying the immunomodulatory sialylated glycans. Future work must employ advanced mass spectrometry-based glycomics and glycoproteomics to: (i) map the exact sialylated structures that define this subtype (e.g., distinguishing between α2–3, α2–6, and α2–8 linkages), (ii) identify the carrier proteins (the “glycoproteome”) that present these immunomodulatory glycans (e.g., PD-L1, MUC1, and integrins), and (iii) determine how this specific “glycocode” changes with disease progression and in response to therapy. Finally, the therapeutic associations identified here are based on transcriptomic signatures and require rigorous experimental evaluation in relevant preclinical models to establish clinical relevance and confirm that these compounds are indeed targeting the tumour glycome.

## 5. Conclusions

In conclusion, this study’s findings suggest that the tumour sialylome is a central organising principle in CRC, defining a biologically and immunologically distinct subset characterised by EMT enrichment, immune exclusion, selective drug resistance programmes, and involvement of the sialic acid–Siglec axis. Our findings reposition glycosylation from a passive structural feature to an active, systems-level determinant of tumour phenotype. They broaden our understanding of cancer heterogeneity and highlight promising translational avenues for the targeting of glycan-dependent tumour–immune interactions. Decoding the “sugar code” is not just an academic exercise in glycobiology; it is essential for understanding the fundamental drivers of cancer progression and for the development of the next generation of precision therapies.

## Figures and Tables

**Table 1:** Summary of significant associations of clinicopathological and molecular features with sialylation after multiple-testing correction.

Feature	Direction of Association	*p*-Value	FDR q-Value
Tumour Location	Higher sialylation in left-sided tumours	0.002	0.004
Histology	Higher sialylation in mucinous adenocarcinoma	<0.001	<0.001
Overall Stage	Higher sialylation in late stage (III-IV)	0.015	0.025
*TP53* Mutation	Higher sialylation in *TP53* mutation-negative tumours	0.022	0.033
*BRAF* Mutation	Higher sialylation in *BRAF* mutation-positive tumours	0.004	0.008
MSI Status	Higher sialylation in MSI tumours	0.009	0.016
Molecular Subtypes	Higher in hypermutated/MSI and mesenchymal/EMT subtypes	0.028	0.04
Aneuploidy	Higher sialylation in low-aneuploidy tumours	<0.001	<0.001
FGA	Higher sialylation in low-FGA tumours	<0.001	<0.001

## Data Availability

All the data explored in this study are domiciled in the Genome Data Commons (https://portal.gdc.cancer.gov/, accessed on 28 January 2026) and the cBioPortal for Cancer Genome (https://www.cbioportal.org/, accessed on 28 January 2026) databases.

## Supplementary Materials

The following supporting information can be downloaded at: https://www.mdpi.com/article/10.3390/biology15090705/s1, Table S1: Gene lists for generation of functional scores. Table S2: Enrichment of glycosylation and ECM-related pathways validate the sialylation dichotomization threshold. Table S3: Clinicopathological and molecular features associated with tumour sialylation status in colorectal cancer. Table S4: Hallmark pathways significantly enriched in the sialylation-high subset of colorectal cancer. Table S5: Top 100 ontology terms enriched in the sialylation-high CRC subset. Table S6: Hallmark pathways significantly enriched in the sialylation-low subset of colorectal cancer. Table S7: Top 100 ontology terms enriched in the sialylation-low CRC subset. Table S8: Enriched Ontology Terms for CD22/CD33-associated gene sets in the sialylation-high subset. Table S9: Lack of Enrichment of Ontology Terms for CD22/CD33-associated gene sets in the sialylation-low subset. Table S10: Drug-Response Signature Enrichment Analysis in Sialylation-High CRC. Table S11: Differential Expression Sialyl-High versus Sialyl-Low.

## Abbreviations

The following abbreviations are used in this manuscript:

GSEA	Gene Set Enrichment Analysis
GOEA	Gene Ontology Enrichment Analysis
DOEA	Drug Ontology Enrichment Analysis
